# Assessment of the Antimicrobial Effectiveness of Herbal Root Canal Irrigants (Propolis, Triphala, and Aloe Vera) and Chlorhexidine Against Enterococcus Faecalis

**DOI:** 10.7759/cureus.41628

**Published:** 2023-07-10

**Authors:** Gondi Durga Bhavani, Tejasree Rathod, Nusrath Parveen, Pudu Tirupathi, Prabhakar Dharavattu, VSSK Sekhar, Devanshi Sharma, SG Anlesteffy

**Affiliations:** 1 Department of Conservative Dentistry and Endodontics, Government Dental College and Hospital, Kadapa, IND; 2 Independent Practitioner, Conservative Dentistry and Endodontics, Hyderabad, IND; 3 Department of Conservative Dentistry and Endodontics, Gitam Dental College, Visakhapatnam, IND; 4 Department of Conservative Dentistry and Endodontics, Sree Sai Dental College and Research Institute, Srikakulam, IND; 5 Prosthodontist, Soham Dental Clinic, Valsad, Gujarat, IND; 6 Periyar University, Department of Microbiology, Salem, IND

**Keywords:** aloe vera, triphala, root canal irrigant, propolis, antibacterial

## Abstract

Background: Complete microbial eradication from the root canal and 3-dimensional obturation of the canal space are necessary for an efficient root canal procedure.

Aim: The current research was conducted to assess the antimicrobial effectiveness of herbal root canal irrigants and Chlorhexidine against *Enterococcus faecalis*.

Materials and methods: The brain heart infusion (BHI) broth was used to grow the *E. faecalis* (ATCC) bacterial culture overnight before it was inoculated onto Mueller-Hinton agar plates. Agar-well diffusion was used to measure antibacterial inhibition. Respective propolis, Triphala, aloe vera, and chlorhexidine irrigants were added to the appropriate wells in agar plates and incubated for 24 hours at 37°C. Each well's bacterial inhibition zone was measured and recorded. Statistics were used to tabulate and analyze the results.

Results: Chlorhexidine indicated the maximum inhibitory zone against *E. faecalis*, subsequently propolis and Triphala, and the lowest by A. vera extract.

Conclusion: Propolis, Triphala, and aloe vera were tested herbal remedies that demonstrated an inhibitory zone against *E. faecalis*. These irrigants are therefore, suitable for use as root canal irrigating solutions.

## Introduction

It is well known that numerous bacterial species contribute significantly to the emergence of pulp and periapical diseases [1,2]. To create the ideal environment for tissue healing, the objective of root canal therapy is to eliminate microorganisms from the root canal [1]. In most cases, *Enterococcus faecalis* is the only source of infection in failed root canals and apical periodontitis [1,3]. It is a gram-positive, facultative anaerobic bacterium [1,2]. It is crucial to the ongoing failure of endodontic therapy [1,4]. Its resistance to intracanal medications is thought to contribute to its virulence [1].

All microorganisms cannot be removed by mechanical methods alone, so appropriate irrigation with intracanal medication is advised [1]. To further reduce microorganisms, using irrigating solutions with potent antimicrobial action is a crucial addition to mechanical preparation [2]. In areas where instrumentation is not available, root canal irrigation aids in the elimination of bacteria. Chlorhexidine (CHX) 2% and sodium hypochlorite (NaOCl) are two examples of chemical root canal irrigants that have been used successfully. The best root canal irrigants should have a pleasant smell and taste, be nontoxic, and be biocompatible [1].

Because of its minimum toxicity and long-lasting broad-spectrum action, chlorhexidine has been used as an irrigant. Chemically speaking, CHX is a synthetic cationic biguanide that gains its potency from its positive charge's interaction with the phosphate group's negative charge on the microbial wall, which changes the cell's osmotic equilibrium [5,6]. It works well as both an irrigant and a medication in endodontics. Both gram-positive and gram-negative microorganisms are inhibited by CHX [7]. CHX has drawbacks such as a bad taste and smell and tissue toxicity. Chlorhexidine has a long-lasting antibacterial effect, but it also discolors teeth and has poor tissue dissolving abilities [5]. NaOCl irritates periapical tissue, triggers allergic reactions, is toxic to tissues, stains tools, is difficult to eliminate smear layers, and has an unpleasant taste and smell [1].

The constant rise in strains that are resistant to antibiotics and the negative side effects of chemical irrigants have prompted a search for substitute herbal remedies. Numerous herbal extracts, including neem, aloe vera, tulsi extracts, *morinda citrifolia*, *curcuma longa*, turmeric, Triphala, proplis, *Salvadora persica* (Miswak), and *Terminalia chebula*, are showing promise for use as endodontic irrigants because of their antimicrobial, anti-inflammatory, and therapeutic properties [1,3,4,8].

Propolis is an organic, biocompatible resinous material that is made from plant materials that honeybees collect. Propolis has antimicrobial and anti-inflammatory properties because it contains flavonoids [9].

The dried and powdered fruits of three medicinal plants, *Terminalia chebula*, *Terminalia bellerica*, and *Emblica officinalis*, are combined in Triphala, an Indian Ayurvedic herbal remedy. Citric acid, which is abundant in it, may help to remove the smear layer and to serve as a chelating agent [4,10]. Excellent antibacterial and anti-inflammatory properties are present in it [10]. 

Aloe vera, which has antibacterial, antifungal, antiviral, anti-inflammatory, and antibacterial properties, is a naturally occurring herbal remedy. *E. faecalis*, *Streptococcus pyogenes*, and *Candida albicans* are all inhibited by the presence of anthrax quinine in aloe vera [5,6].

However, there isn't much information or proof on the comparison of propolis, Triphala, and aloe vera together for antibacterial properties in endodontics. Thus, the goal of the present research was to assess the antimicrobial effectiveness of herbal root canal irrigants (propolis, Triphala, and aloe vera) and chlorhexidine (a positive control) against *E. faecalis*.

## Materials and methods

The current research was carried out in the conservative dentistry and endodontics departments.

Propolis irrigation solution was made by diluting a 33% concentration of propolis, which was commercially available (Hi-Tech Natural Products India Ltd., Delhi, India), by a 2:1 ratio with warm saline [8].

Triphala preparation: To make an irrigation solution with a 5 mg/ml concentration, Triphala powder (IMPCOPS Ltd., Chennai, India) was dissolved in 10% dimethylsulfoxide (SD Fine Chemicals, Chennai, India).

Aloe vera extract preparation: Fresh aloe vera plants' leaves were harvested, and the pulp was manually extracted. Following the addition of 2.5 mL of chloroform to 1000 mL of purified water (Indian Pharmacopoeia) to the aloe vera plant pulp, the mixture was filtered using a double-filter paper. The supernatant was then centrifuged at 8,000 rpm for 40 minutes to extract the extract. Using distilled water, serial dilutions of 5/95, 25/75, 50/50, and 100 mL (volume/volume) were made to obtain 5%, 25%, 50%, and 100% concentrations, respectively, for the evaluation of antimicrobial activity against *E. faecalis* from the concentrated solutions made from all the aforementioned ingredients. On the same day that the extracts were prepared, antimicrobial testing was conducted.

Microbiological analysis: For intracanal irrigation, 40 samples were categorized into four groups (one positive control and three test groups) with 10 samples each: Group I: Chlorhexidine as the positive control group, and test groups; Group II: Propolis, Group III: Triphala, and Group IV: Aloe vera. The microbiological laboratory served as the location for the microbiological investigation.

A pure culture of the test strain, E. faecalis ATCC 29212, was created on Mueller Hinton Agar (HiMedia Laboratory Private Limited, Mumbai, Maharashtra, India). Then, using a Shimadzu UV 2400 PC, Tokyo, Japan, it was spectrophotometrically adjusted to an optical density of 560 nm, correlating to the turbidity of the McFarland 0.5 scale [1.5x108 colony]. The antibacterial inhibition zones around propolis, Triphala, aloe vera, and chlorhexidine medications were identified using the agar disc diffusion method. The whole process was conducted using the asepsis method. Each medication was placed in the appropriate well of the BHI agar plates, which had been prepared. The plates were kept in a 37°C incubator for 24 hours. The bacterial inhibition zone around each well was measured after the plates had been incubated [1].

The tabulated data were statistically evaluated using IBM Corp. Released 2013. IBM SPSS Statistics for Windows, Version 22.0. Armonk, NY: IBM Corp., with an ANOVA test followed by a post hoc Tukey test at P<0.05.

## Results

**Figure 1:**
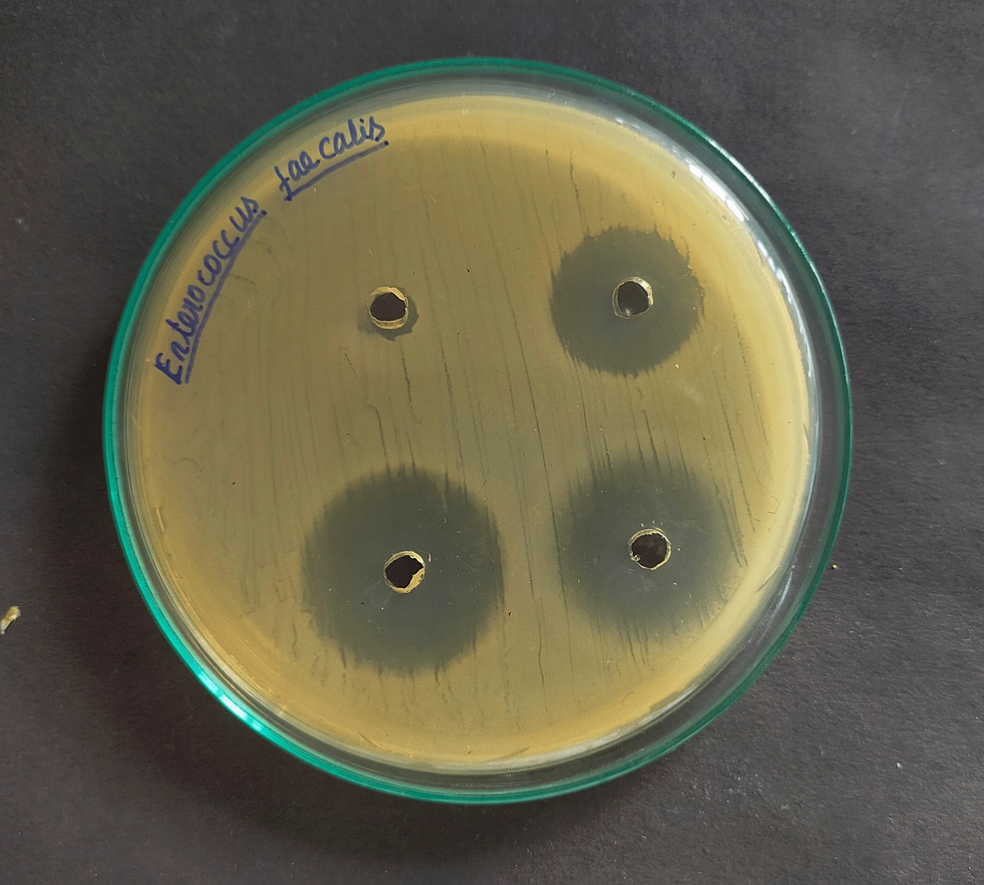
Inhibitory zone (mm) of 2% chlorhexidine against Enterococcus Faecalis

**Figure 2:**
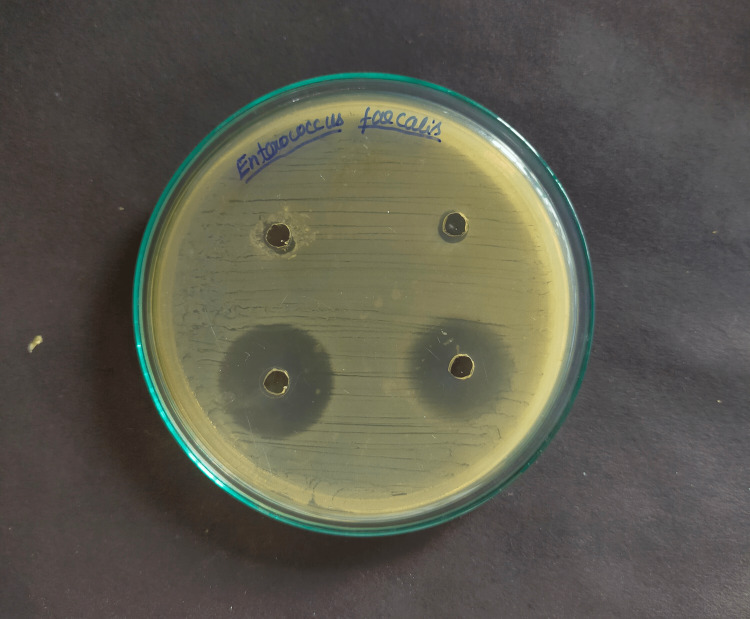
Inhibitory zone (mm) of propolis against Enterococcus Faecalis

**Figure 3:**
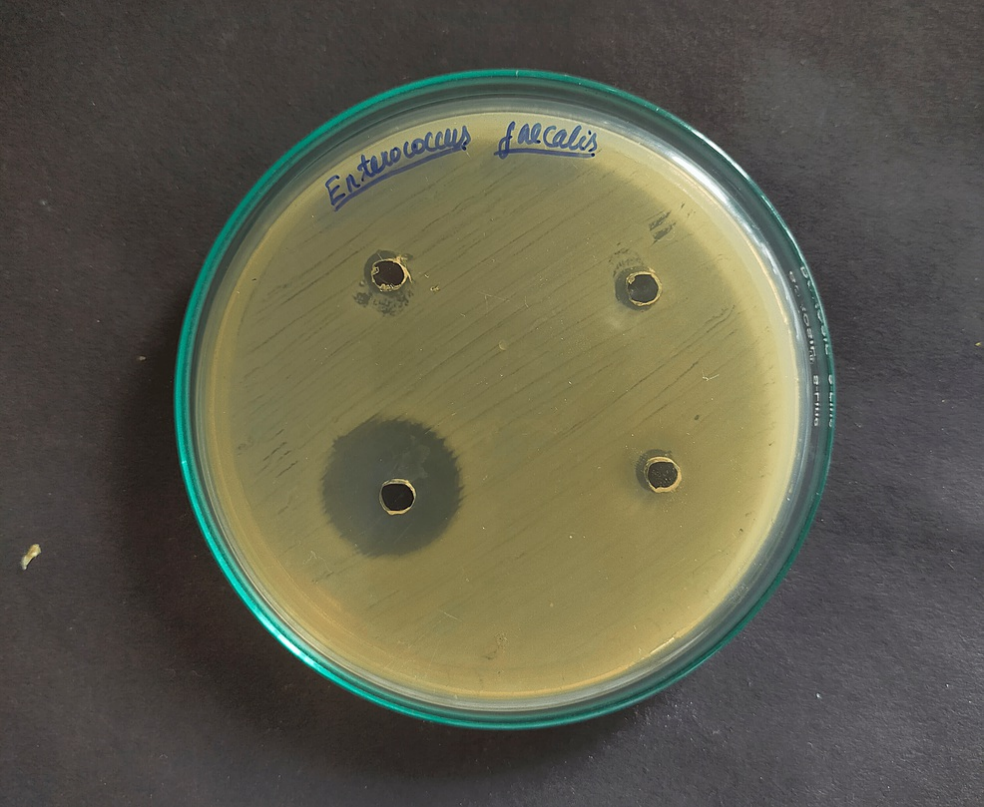
Inhibitory zone (mm) of Triphala against Enterococcus Faecalis

**Figure 4:**
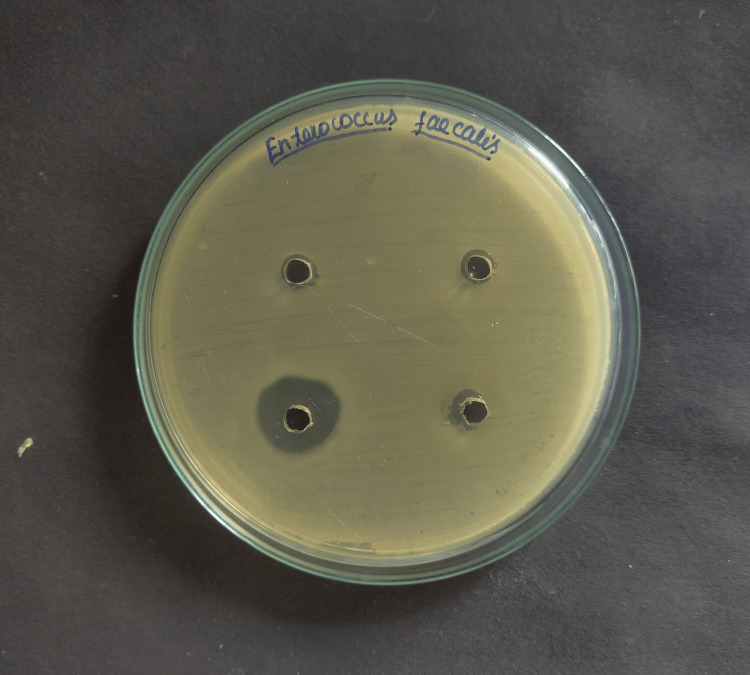
Inhibitory zone (mm) of aloe vera against Enterococcus Faecalis

For measuring the zones of inhibition around each of the four wells, the Mueller-Hinton Agar test was used to assess the antibacterial activity against *E. Faecalis* with three different herbal medicaments and chlorhexidine (Figures 1-4). 

Each sample's mean zone of inhibition was calculated, and the results are shown in Table 1. Results were recorded based on the diameter of the zones.

Positive control, chlorhexidine 2% (29.57mm) had the maximum mean inhibitory zone against E. faecalis, followed by propolis (23.45mm), Triphala (19.32mm), and A. vera extract (12.67mm) (Table 1). And the difference was statistically significant because the p-value was < 0.05. The mean zone of inhibition in descending order for each drug was found to be Chlorhexidine 2% >propolis > Triphala > A. vera extract.

**Table 1:** Inhibitory zone (mm) against Enterococcus Faecalis by various root canal irrigants SD: Standard deviation, SE: Standard error, p<0.05

Group	N	Mean± SD zone of inhibition in mm	SE	p-value
Group I: 2% Chlorhexidine	10	29.57±1.264	0.324	0.001
Group II: Propolis	10	23.45±1.129	0.275	
Group III: Triphala	10	19.32±1.035	0.235	
Group IV: Aleo vera	10	12.67±1.078	0.175	

The intergroup comparison of herbal irrigants over chlorhexidine is highly significant (p<0.001) (Table 2).

**Table 2:** Intergroup comparison of inhibitory zones against E. faecalis Test used: post hoc Tukey test, p<0.05

Inter Group comparison		Difference in Mean± SD zone of inhibition in mm	p-value
Group I: 2% Chlorhexidine	Group II	6.12±0.135	<0.05
	Group III	10.25±0.229	<0.001*
	Group IV	16.9±0.186	<0.001*
Group II: Propolis	Group I	-6.12±0.135	<0.05
	Group III	4.13±0.094	<0.001*
	Group IV	10.78±0.051	<0.001*
Group IIII: Triphala	Group I	-10.25±0.229	<0.001*
	Group II	-4.13±0.094	<0.001*
	Group IV	6.65±0.043	<0.001*
Group IV: Aloe vera	Group I	-16.9±0.186	<0.001*
	Group II	-10.78±0.051	<0.001*
	Group III	-6.65±0.043	<0.001*

## Discussion

The long-term success of nonsurgical endodontics depends on thorough debridement and efficient disinfection of the root canal space [6]. In the tiny canals of apical ramifications or in the space between the root filling and canal wall, bacteria like *E. faecalis* can survive. *E. faecalis* strains can endure in an environment with few nutrients and reduced commensality with other bacteria for at least 6 to 12 months. Chemicals like calcium hydroxide are not able to harm *E. faecalis* at all in such conditions [5].

Since they have high antimicrobial activity, natural or herbal products have been used for thousands of years in dental and medical procedures [6]. In the current study, chlorhexidine had the best antimicrobial effectiveness against *E. faecalis*, followed by propolis, Triphala, and aloe vera. Because it is the most common species found in failed endodontic cases. *Enterococcus faecalis*, a gram-positive facultative anaerobe, was selected as the study's main test organism. In the current research, the BHI culture method was used to identify zones of inhibition. Since the culture technique is one of the most reliable ways to identify live bacteria, it was used to find *E. faecalis* inside the root canal [2].

Aloe vera, *Morinda citrifolia*, and *Azadirachta indica* extract all demonstrated an inhibitory zone against *E. faecalis*, and they can all be used as root canal irrigating solutions, according to Babaji et al.'s assessment of the antimicrobial effectiveness of herbal root canal irrigants with sodium hypochlorite (NaOCl) [1].

Vinoth Kumar et al. assessed the antimicrobial effectiveness of different herbal extracts such as *Azadiracta indica* (AI), *Curcuma longa* (CL), *Myristica fragrans* (MF), *Aloe barbadensis* (AV), and *Terminalia chebula* (TC) as endodontic irrigants against *E. faecalis* and *Candida albicans*. They concluded that neem leaf extract has considerable antimicrobial efficiency against *E. faecalis* [3].

Propolis displayed the highest zone of inhibition in the current study compared to all other tested herbal groups, and this may be because propolis contains significant amounts of pharmacologically active constituents like flavonoids and phenolics. Flavonoids are among the main polyphenols in propolis. The antibacterial effect of flavonoids occurs by inhibiting the synthesis of DNA or RNA in bacteria [11]. Flavonoids are thought to be responsible for most of propolis' biological activity. Propolis ethanol extract has effective antimicrobial properties for use in endodontic procedures [4]. Similar to our findings, Saxena et al., Ozant Oncag et al., and Ferreira et al. found that propolis had good in vitro antibacterial activity against *E. faecalis* but was less effective than chlorhexidine or sodium hypochlorite in use as a root canal irrigant [4,12,13]. The conclusions concur with our findings. According to Daga et al.’s findings, propolis, neem, and miswak were the next best irrigants, followed by sodium hypochlorite [8]. When compared to 2% CHX, Rathee et al. found that herbal products (neem and tulsi extract) had significant antimicrobial activity in treating primary endodontic infections [14]. Saxena et al. compared the antimicrobial activity of 2.5% sodium hypochlorite and five herbal extracts, including propolis, Triphala, *C. longa*, and MC, against *Enterococcus faecalis*. They concluded that, among all herbal extracts, propolis displayed the highest zone of inhibition [4]. 

Citric acid, which is abundant in Triphala, may help to remove the smear layer and act as a chelating agent, making it a useful complement to sodium hypochlorite for root canal irrigation [4]. Triphala components adsorb well on bacterial cell surfaces and result in protein denaturation [15]. As a result, we discovered a higher zone of inhibition with Triphala in comparison to aloe vera. Triphala, as a root canal irrigant, has been shown to have a greater antimicrobial effect than sterile saline in reducing microbial flora, according to Divya and Sujatha [10]. Sodium hypochlorite 5%, Triphala, and 2% chlorhexidine gluconate solutions all demonstrated maximum and comparable antibacterial efficacy against *E. faecalis*, according to Srikumar et al. [16].

*Tridax procumbens* and *Aegle Marmelos* also demonstrated statistically significant antibacterial activity, according to Surender et al. [17]. Nisin was found to have an action that was comparable to CHX, according to Nirmala et al.'s conclusion [7].

Aloe vera has an inhibitory zone against *E. faecalis*, according to Nagaveni et al. [5]. Pomegranate, garlic, and aloe Vera, according to Gandhi et al., had a lower mean zone of inhibition than 0.2% chlorhexidine [6]. Contrary to our findings, Karkare et al. found that aloe vera displayed the maximum inhibitory zone against *E. faecalis* [18]. Our findings concur with those of Babaji et al. and Jose et al., who demonstrated that sodium hypochlorite, or CHX, has greater antimicrobial effectiveness than aloe vera [1,19].

Triphala and propolis had stronger antimicrobial effects, which may be related to their antimicrobial properties. Aloe vera extract reduced the inhibition zone, which may be related to the use of plants with varying chemical make-ups from various geographic locations. Weather changes where the plant was grown and where it was made may be the probable cause of Aloe vera's decreased antimicrobial efficacy in the present study [1,19].

In the current study, chlorhexidine had the highest antimicrobial effectiveness against *E. faecalis*, followed by propolis, Triphala, and aloe vera. Saxena et al. compared the antimicrobial activity of 2.5% sodium hypochlorite with propolis, Triphala, *C. longa*, and MC against *Enterococcus faecalis*. They found a higher inhibitory zone with aloe vera compared to Triphala [NaOCl (22.0 ± 1.53) > propolis (12.2 ± 1.53) > AI (08.8 ± 1.60) > Triphala (05.1 ± 0.94)] in contrast to our study. [4]. Nagaveni et al. compared aloe vera alone with chlorhexidine against *E. faecalis*, according to [5]. Srikumar et al. compared sodium hypochlorite 5%, Triphala, and 2% chlorhexidine gluconate solutions against *E. faecalis* [16]. Our study is unique compared to reported earlier studies since we compared propolis, Triphala, and aloe vera with chlorhexidine. This comparison has not yet been reported. Our study comparison stated that propolis had a higher inhibitory zone and was comparable to chlorhexidine, followed by Triphala and aloe vera. The intergroup comparison is highly significant. These findings guide the clinician in choosing herbal alternative irrigants.

The main benefits of using herbal substitutes are their accessibility, affordability, low toxicity, and longer shelf life. Additionally, they provide significant therapeutic advantages and are more affordable than synthetic alternatives [6]. To confirm our findings, additional research is required.

Limitation of the study: The present study was an in vitro study with a limited sample size. A major disadvantage of herbal extracts is the need for fresh preparation and modification in taste for acceptability.

## Conclusions

Chlorhexidine is considered as a strong antimicrobial agent. Alternative herbal medications have been tried as a result of sodium hypochlorite and chlorhexidine's side effects. The present study showed that chlorhexidine, followed by propolis, Triphala, and aloe vera, developed an antimicrobial inhibition zone against *E. faecalis*. Tested herbal substitutes have low toxicity, increased shelf life, and are easily accessible. As a result, these herbal irrigants are also appropriate to use in root canals. Since the majority of studies are ex vivo, there is little information available regarding the efficiency, safety, and higher quality of these products for use in dentistry; hence additional in vivo studies must be conducted to confirm the result of the study.
